# Laparoscopic common bile duct exploration with primary closure could be safely performed among elderly patients with choledocholithiasis

**DOI:** 10.1186/s12877-023-04149-w

**Published:** 2023-08-11

**Authors:** Lili Fan, Yan Wang, Meilong Wu, Tianchong Wu, Lingna Deng, Yawei Wang, Linsen Liu, Tailai An

**Affiliations:** 1 Department of Geriatric Medicine, Shenzhen Traditional Chinese Medicine Hospital, Fuhua Road 1, Futian District, Shenzhen, 518033 Guangdong China; 2 Department of Radiology, Shenzhen People’s Hospital, Dongmen Road 1017, Luohu District, Shenzhen, 518020 Guangdong China; 3 Department of Hepatobiliary and Pancreatic Surgery, Shenzhen People’s Hospital, Dongmen Road 1017, Luohu District, Shenzhen, 518020 Guangdong China; 4 Department of Pathology, Qingyuan People’s Hospital, Yinquan Road B24, Qingcheng District, Qingyuan, 511518 Guangdong China; 5 The First Department of Surgery, Shenzhen Traditional Chinese Medicine Hospital, Fuhua Road 1, Futian District, Shenzhen, 518033 Guangdong China

**Keywords:** Choledocholithiasis, Laparoscopic common bile duct exploration, Primary closure of CBD, Bile leakage, Elderly patients

## Abstract

**Background:**

For patients with choledocholithiasis, laparoscopic common bile duct exploration (LCBDE) is preferred over open surgery. Whether primary closure of the common bile duct (CBD) should be performed upon completion of choledochotomy remains unclear, and the corresponding indications for primary closure of the common bile duct have yet to be fully identified. This study was performed to evaluate the safety and feasibility of primary closure of CBD among elderly patients (≥ 70 years) after LCBDE.

**Methods:**

Patients with choledocholithiasis who had undergone LCBDE with primary closure of the CBD between July 2014 and December 2020 were retrospectively reviewed. Included patients were assigned into two groups (Group A: ≥70 years and Group B: <70 years) according to age. Group A was compared with Group B in terms of preoperative characteristics, intraoperative results and postoperative outcomes.

**Results:**

The mean operative time for Group A was 176.59 min (± 68.950), while the mean operative time for Group B was 167.64 min (± 69.635) (P = 0.324). The mean hospital stay after surgery for Group A was 8.43 days (± 4.440), while that for Group B was 8.30 days (± 5.203) (P = 0.849). Three patients in Group A experienced bile leakage, while bile leakage occurred in 10 patients in Group B (3.8% vs. 4.5%, P = 0.781). Group A was not significantly different from Group B in terms of postoperative complications and 30-day mortality except pneumonia (P = 0.016), acute cardiovascular event (P = 0.005) and ICU observation (P = 0.037). After a median follow-up time of 60 months, 2 patients in Group A and 2 patients in Group B experienced stone recurrence (2.5% vs. 0.9%, P = 0.612). One patient in Group A experienced stenosis of the CBD, while stenosis of the CBD occurred in 5 patients in Group B (1.3% vs. 2.2%, P = 0.937).

**Conclusions:**

Primary closure of CBD upon completion of LCBDE could be safely performed among patients ≥ 70 years.

## Background

Choledocholithiasis is a common disease. The incidence of cholelithiasis ranges from 5 to 15%, of which 5-30% of cases are combined with choledocholithiasis [1]. Choledocholithiasis is the result of stones expelled from the intrahepatic bile duct or gallbladder. Despite the fact that some stones can pass into the duodenum via major duodenal papilla, some stones may not pass through the CBD so smoothly due to large sizes, and obstruction caused by large stones may subsequently lead to acute cholangitis or acute pancreatitis [2]. Among elderly patients, choledocholithiasis is quite commonly diagnosed. According to some previous studies, the incidence of choledocholithiasis increased with age [3–5]. An increased incidence of choledocholithiasis means more hepatobiliary surgeries for patients with high risks.

Treatment choices for patients with choledocholithiasis vary significantly, including laparotomy, laparoscopic or robotic surgery, endoscopic retrograde cholangiopancreatography (ERCP) and percutaneous intervention [6]. For patients suffering from choledocholithiasis, laparoscopic common bile duct exploration (LCBDE) can be safely performed [7–9]. Additionally, primary closure of the CBD without inserting a T-tube drainage tube has been advocated as a feasible alternative to T-tube insertion after LCBDE, which may further significantly reduce surgical trauma and remarkably increase quality of life after surgery [10–12]. At our center, laparoscopic common bile duct exploration has become a mature procedure and it would be performed if no remarkable contraindications have been encountered. However, besides laparoscopic common bile duct exploration with primary closure, other procedures have also been performed at our center. Of all common bile duct surgeries, about 3.2% were open common bile duct exploration where laparoscopic operations had not been tried, 84.7% were laparoscopic common bile duct exploration with primary closure, 10.6% were laparoscopic common bile duct exploration with T tube drainage and 1.5% were open common bile duct exploration converted from laparoscopic surgeries.

The prevalence of comorbidities significantly increases with increasing age. However, studies investigating the feasibility and safety of primary closure of CBD among elderly patients with choledocholithiasis are still needed. The present study was performed to further assess the safety and feasibility of primary closure of CBD on completion of LCBDE among patients ≥ 70 years of age in comparison with those < 70 years of age.

## Materials and methods

### Patients and ethical approval

Data from patients with choledocholithiasis who had undergone primary closure of the CBD without inserting a T drainage tube upon completion of LCBDE at the Department of Hepatobiliary and Pancreatic Surgery, Shenzhen People’s Hospital between July 2014 and December 2020 was retrospectively reviewed. The inclusion criteria were as follows: laparoscopic common bile duct with primary closure and complete and searchable medical records. Whereas, the following criteria were adopted as the exclusion criteria: emergent operation, concomitant acute suppurative cholecystitis or cholangitis, concurrent hepatolithiasis, Mirizzi syndrome, carcinoma of gallbladder or the bile duct, age < 16 years and hematological diseases predisposing significantly higher risk of cholelithiasis. Diagnoses of choledocholithiasis were made by combining symptoms, physical signs, and imaging examination results. Both magnetic resonance cholangiopancreatography (MRCP) and abdominal ultrasonography were routinely performed, while computed tomography (CT) was performed as an alternative to MRCP for patients who could not undergo MRCP due to different contraindications. The diameter of the CBD and characteristics of stones within the CBD (number and diameter) were identified by the aforementioned imaging examinations. Informed consent in written form was acquired from each patient, and the present study was approved by The Ethical Committee, Shenzhen People’s Hospital. Patients were assigned into the elderly group (Group A, ≥ 70 years) and the younger group (Group B, < 70 years). All procedures were performed under general anesthesia by two to three surgeons. Consistent with standards from some previous studies [3, 13–16], the following were our standards for primary closure of CBD: diameter of CBD ≥ 8 mm, both stones in CBD and within intrahepatic bile duct thoroughly extracted, mild inflammation of CBD wall, patent distal CBD and major duodenal papilla, well functional Oddi’s sphincter, and without severe injuries to supplying vessels of CBD. In theory, distance between incision and supplying vessels (at 3 o’clock and 9 o’clock of common bile duct) and frequency of using electrocoagulation are two main factors contributing to injuries to supplying vessels. Usually, the incision was made where CBD, cystic duct and common hepatic duct converged since there were significantly less dense supplying vessels. However, when the incision was made too near to the supplying vessels (at 3 o’clock and 9 o’clock of CBD ), severe injuries to these supplying vessels and much blood loss would occur. Additionally, electrocoagulation would further aggravate these injuries to supplying vessels. If distance between incision and supplying vessels of CBD was appropriate and no significant blood loss occurred, then no severe injuries to supplying vessels were defined. Contrarily, if distance between incision and supplying vessels of CBD was too short and significant blood loss was recorded, then moderate injuries to supplying vessels of CBD were defined. On basis of moderate injuries, if electrocoagulation had to be adopted to control blood loss, then severe injuries to supplying vessels were defined. However, this method was not quantitative and novel ways quantitatively assessing injuries to supplying vessels of CBD should be developed.

### Operational procedures

**Fig. 1:**
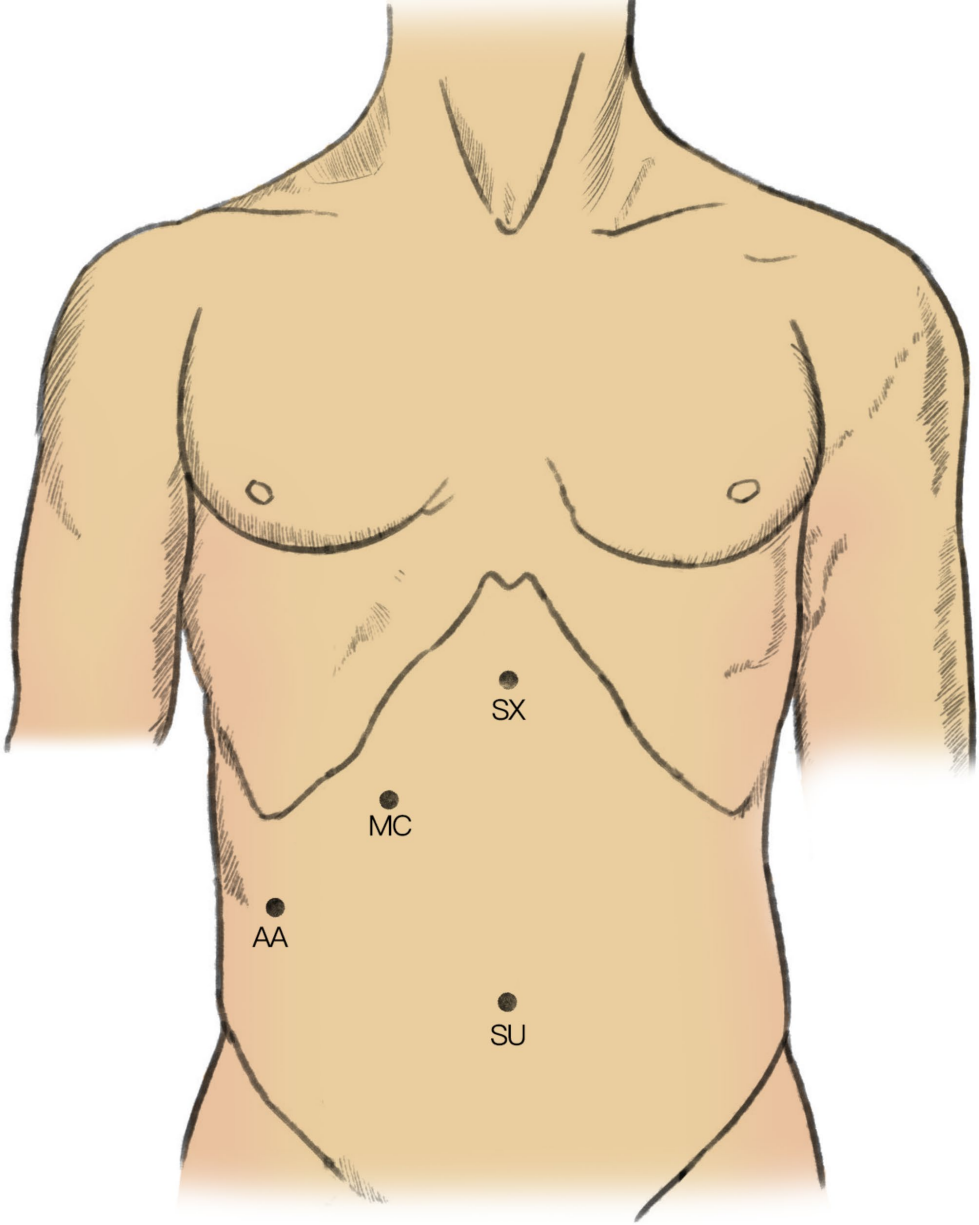
Positions of trocars

**Fig. 2:**
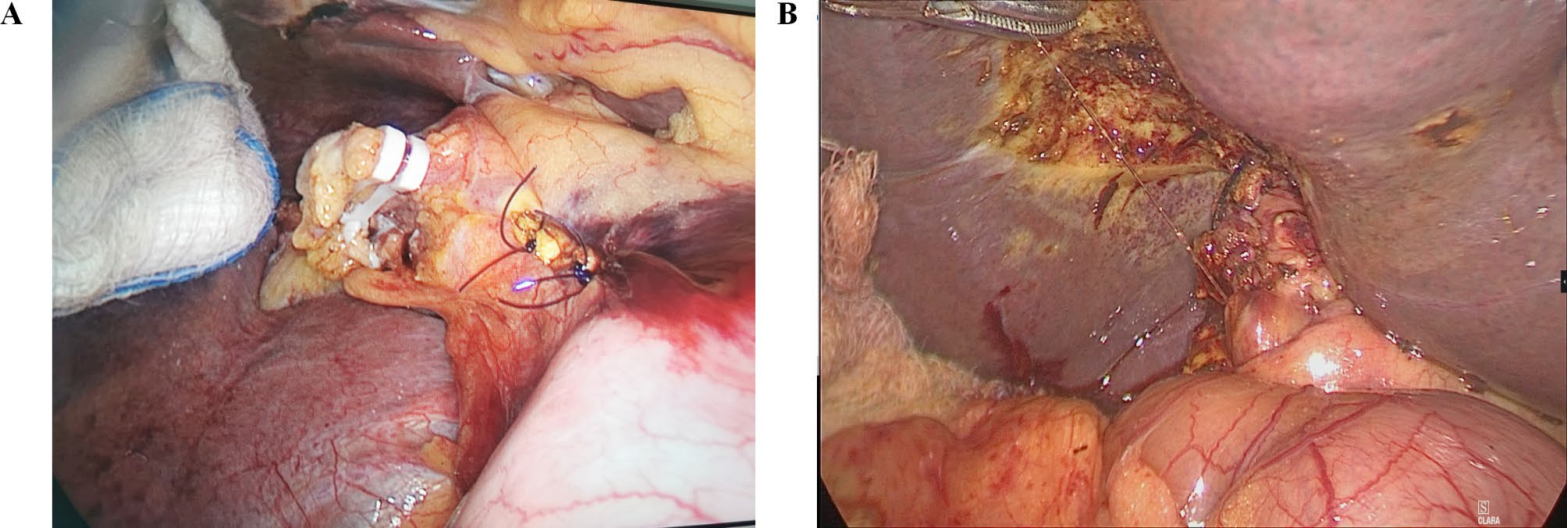
** A**: A representative image demonstrating laparoscopic common bile duct exploration via the common bile duct with primary closure. **B**: A representative image demonstrating laparoscopic common bile duct exploration via the cystic duct with primary closure

Initially, laparoscopic cholecystectomy (LC) was accomplished by a three-port configuration. First, the subumbilical trocar for inserting the camera was established. Then, under directional vision, the epigastric port (10 mm) and the right midclavicular port (5 mm) were established. The surgeons in charge would decide whether cystic bile duct would be preserved before common bile duct exploration. Most surgeons would not preserve the cystic bile duct while other surgeons would preserve the cystic bile duct as a traction for common bile duct exploration. If the cystic bile duct was not to be preserved, gallbladder would be then removed. If the cystic bile duct was to be preserved, gallbladder would be removed after common bile duct exploration. The gallbladder was removed using an electrotome or a harmonic dissector (Ultracision harmonic scalpel; Ethicon Endo-surgery, Cincinnati, OH, USA). After LC, another 5-mm trocar was established to serve as an assistive port. The distribution of the four trocars mentioned above is shown in Fig. 1. For patients who had already undergone cholecystectomy, LCBDE was directly performed using the four-port configuration described above. For patients with a cystic duct less than 5 mm, LCBDE was accomplished via the transcholedochal approach as described in some previous studies [3, 17]. The anterior surface of the CBD was cut open longitudinally using surgical scissors or electric hooks, and then according to the sizes of the stones, we performed choledochotomy via an incision between 8 and 12 mm. Then, we performed CBD exploration by inserting a 5 mm choledochoscope (Olympus, Tokyo, Japan). In most cases, the 5 mm choledochoscope could be inserted into the right and left hepatic ducts, and secondary intrahepatic bile ducts could be observed under the choledochoscope. When the biliary system was remarkably dilated, the choledochoscope could even be inserted into the secondary intrahepatic bile duct. Both stones in the CBD and visible calculi within the intrahepatic bile duct were extracted using suction, basket or electrohydraulic lithotripsy. In addition to being used to extract stones, the choledochoscope was also adopted to observe the patency of Oddi’s sphincter and distal CBD. Patency and status of Oddi’s sphincter and distal CBD were carefully inspected to confirm whether the outlet of CBD was obstructed. Additionally, intraoperative cholangiogram would also be performed to make sure that no stones had been left and Oddi’s sphincter and distal CBD were patent. Intraoperative cholangiogram was performed prior to closure of the choledochotomy. For transcystic exploration, transcystic cholangiogram would be performed. In order to prevent leakage of contrast agent, clips were used to secure the cystic duct and then a catheter was inserted into the common bile duct. Then cholangiogram was performed by injecting contrast agent into the common bile duct. For transcholedochal exploration, transcholedochal cholangiogram would be performed. Similarly, clips were used to secure the incision on CBD and then a catheter was inserted into the common bile duct. Then cholangiogram was performed by injecting contrast agent into the common bile duct. Primary closure of the CBD was performed only if the following conditions were met: good contraction and favorable peristalsis of Oddi’s sphincter could be directly observed under the choledochoscope, and the choledochoscope could be successfully inserted into the duodenum without remarkable resistance; otherwise, a 5-Fr ureteric catheter was inserted through the choledochoscope into the lower part of the CBD and could smoothly enter the duodenum via Oddi’s sphincter. When the aforementioned conditions were fulfilled, the incision on the CBD was continuously closed using a 5 − 0 polydioxanone suture. Representative images of LCBDE via CBD and primary closure are shown in Fig. 2A.

For patients with cystic ducts larger than 5 mm, a transcystic approach was adopted to accomplish LCBDE. Initially, the cystic duct was dilated using a balloon dilator. When dilating the cystic duct using a balloon dilator was impossible, we then made a longitudinal incision on the cystic duct. Then, the choledochoscope was inserted into the CBD via the cystic duct. After extracting stones within bile ducts, we then closed the cystic duct using sutures or clips. Representative images of LCBDE via the cystic duct and primary closure are shown in Fig. 2B.

After the completion of surgery, patients were transferred back to the general ward or intensive care unit (ICU) considering vital signs during surgery, recovery of autonomous respiration and consciousness. For all patients, a rubber drainage tube was routinely placed into the subhepatic area. Two to three days after surgery, we removed the drainage tube when less than 20 ml/day was drained out. When the following standards were met, patients were discharged: stable vital signs and normal laboratory tests, being able to autonomously accomplish off-bed activities, a good appetite and normal excretion, without surgical site infection or poorly healed incision.

### Follow-up

Unless otherwise contraindicated, all the patients were instructed to attend a postoperative follow-up lasting 60 months every six months. Consistent with previous studies, stones identified 6 months after the complete removal of primary stones were recorded as recurrence of choledocholithiasis [1, 18, 19]. During the subsequent follow-up, clinical manifestations such as epigastric pain, abnormal liver function test, obstructive jaundice and cholangitis would suggest CBD stenosis. Then CT or/and MRI + MRCP would be performed to assess patency of CBD. Sometimes endoscopic retrograde cholangiopancreatography (ERCP) would be performed to verify CBD stenosis.

### Statistical analysis

Continuous variables are presented as the mean ± standard deviation (SD), while categorical variables are presented as percentages. Comparisons of baseline characteristics, preoperative factors, operation-related variables and postoperative morbidity were accomplished by Student’s t test, Fisher’s exact test or chi-squared analysis when appropriate. Statistical Package for the Social Sciences (SPSS, version 22.0) was used to perform statistical analyses in this study. Comparisons with P values less than 0.05 were considered statistically significant.

## Results

### Baseline characteristics and comorbidities

The two groups were compared regarding baseline characteristics, including age, sex, body mass index (BMI), obstructive jaundice, acute cholangitis, history of pancreatitis, history of abdominal surgery, history of cholecystectomy, diameter of CBD, number of CBD stones, and maximum diameter of stones, revealing that Group A had a more common history of cholecystectomy (P = 0.023) (Table 1). Additionally, Group A was also compared with Group B in terms of comorbidities and ASA score, demonstrating that significantly more patients in Group A had hypertension (P < 0.001), diabetes mellitus (P = 0.002), cardiovascular diseases (P < 0.001) and higher ASA scores (P < 0.001) (Table 2).

**Table 1:** Comparisons between Group A and Group B regarding demographics and clinical factors

Characteristics	No.	A	B	χ^2^/t	P
	(N = 303)	(N = 80)	(N = 223)	Value	Value
Gender				0.175	0.676
Male	150(49.5%)	38(47.5%)	112(50.2%)		
Female	153(50.5%)	42(52.5%)	111(49.8%)		
BMI				1.440	0.696
≤ 18.4(kg/m^2^)	21(6.9%)	6(7.5%)	15(6.7%)		
18.5–23.9(kg/m^2^)	161(53.1%)	45(56.3%)	116(52.0%)		
24-27.9(kg/m^2^)	97(32.0%)	25(31.2%)	72(32.3%)		
≥ 28(kg/m^2^)	24(8.0%)	4(5.0%)	20(9.0%)		
Obstructive jaundice				2.103	0.147
No	75(24.8%)	15(18.8%)	60(26.9%)		
Yes	228(75.2%)	65(81.2%)	163(73.1%)		
Acute cholangitis				2.258	0.133
No	143(47.2%)	32(40.0%)	111(49.8%)		
Yes	160(52.8%)	48(60.0%)	112(50.2%)		
History of pancreatitis				0.001	0.972
No	273(90.0%)	72(90.0%)	201(90.1%)		
Yes	30(10.0%)	8(10.0%)	22(9.9%)		
History of abdominal surgery				0.371	0.542
No	231(76.2%)	59(73.8%)	172(77.1%)		
Yes	72(23.8%)	21(26.2%)	51(22.9%)		
History of cholecystectomy				5.161	**0.023**
No	286(94.4%)	71(88.8%)	215(96.4%)		
Yes	17(5.6%)	9(11.2%)	8(3.6%)		
Diameter of CBD				3.603	0.058
≤ 8 mm	37(12.2%)	5(6.3%)	32(14.3%)		
>8 mm	266(87.8%)	75(93.7%)	191(85.7%)		
Solitary/multiple stones				3.661	0.056
Solitary	149(49.2%)	32(40.0%)	117(52.5%)		
Multiple	154(50.8%)	48(60.0%)	106(47.5%)		
Maximum diameter of stones (mm)	7.87 ± 4.786	8.39 ± 4.462	7.68 ± 4.893	-1.132	0.258

**Table 2:** Comparisons between Group A and Group B regarding preoperative risk factors

Characteristics	No.	A	B	χ^2^/t	P
	(N = 303)	(N = 80)	(N = 223)	Value	Value
Hypertension				32.217	**< 0.001**
No	221(72.9%)	39(48.8%)	182(81.6%)		
Yes	82(27.1%)	41(51.2%)	41(18.4%)		
Diabetes mellitus				9.880	**0.002**
No	276(91.1%)	66(82.5%)	210(94.2%)		
Yes	27(8.9%)	14(17.5%)	13(5.8%)		
Cardiovascular disease				24.730	**< 0.001**
No	276(91.1%)	62(77.5%)	214(96.0%)		
Yes	27(8.9%)	18(22.5%)	9(4.0%)		
Pulmonary disease				1.273	0.259
No	295(97.4%)	76(95.0%)	219(98.2%)		
Yes	8(2.6%)	4(5.0%)	4(1.8%)		
Renal insufficiency					
No	299(98.7%)	79(98.8%)	220(98.7%)	0.004	0.949
Yes	4(1.3%)	1(1.2%)	3(1.3%)		
ASA score	1.50 ± 0.645	2.00 ± 0.656	1.32 ± 0.540	-9.071	**< 0.001**
1	173(57.1%)	15(18.8%)	158(70.9%)	69.138	**< 0.001**
2	111(36.6%)	52(65.1%)	59(26.5%)		
3	16(5.3%)	11(13.8%)	5(2.2%)		
4	3(1.0%)	2(2.5%)	1(0.4%)		

### Imaging examinations identifying choledocholithiasis

At our center, patients suspected with common bile duct stones were routinely recommended to undergo CT and MRI + MRCP unless otherwise contraindicated. However, due to different clinical situations, not all the patients could undergo both CT and MRI + MRCP. We searched the picture archiving and communication system(PACS) and medical record system. It was revealed that 101 patients were confirmed with choledocholithiasis by CT (including 89 ones having not undergone MRI + MRCP, 12 ones having undergone MRI + MRCP but not confirmed with choledocholithiasis by MRI + MRCP), 97 patients were confirmed with choledocholithiasis by MRI + MRCP (including 57 ones having undergone CT and 40 ones having undergone CT but not confirmed with choledocholithiasis by CT) and 105 patients were confirmed with choledocholithiasis by both CT and MRI + MRCP.

### Intraoperative variables

Eight patients in Group A underwent transcystic exploration of the CBD, while 72 patients underwent transcholedochal surgery; for patients in Group B, 17 patients underwent transcystic exploration of the CBD, while 206 patients underwent transcholedochal surgery (P = 0.507). The mean operative time for Group A was 176.59 min (± 68.950), while the average operative time for Group B was 167.64 min (± 69.635) (P = 0.324). Group A was not significantly different from Group B in terms of estimated blood loss (P = 0.075). Due to major hemorrhage, 3 patients in Group A needed blood transfusion, while 1 in Group B needed intraoperative blood transfusion (P = 0.099). Six patients in Group A were transferred to the ICU for recovery and early postoperative care after surgery, which occurred in 4 patients in Group B (P = 0.037). The mean hospital stay after surgery for Group A was 8.43 days (± 4.440), while that for Group B was 8.30 days (± 5.203) (P = 0.849).

### Postoperative complications

Group A was not significantly different from Group B in terms of most postoperative complications except for a higher incidence of pneumonia (P = 0.016) and acute cardiovascular events (P = 0.005) for Group A (Table 3). In Group A, remnant stones occurred in 4 patients, and 9 patients in Group B experienced remnant stones (5.0% vs. 4.0%, P = 0.965). Presentations of remnant stones were persistent upper abdominal pain and fever. Remnant stones were confirmed by MRCP. Remnant stones within the CBD were extracted by endoscopic sphincterotomy (EST).

**Table 3:** Comparisons between Group A and Group B regarding postoperative results and follow-up outcomes

Characteristics	No.	A	B	χ^2^/t	P
	(N = 303)	(N = 80)	(N = 223)	Value	Value
Operation type				0.439	0.507
Transcystic exploration	25(8.3%)	8(10%)	17(7.6%)		
Transcholedochal exploration	278(91.7%)	72(90%)	206(92.4%)		
Operative time (min)	170.00 ± 69.453	176.59 ± 68.950	167.64 ± 69.635	-0.988	0.324
Estimated blood loss (mL)				3.168	0.075
<100 ml	273(90.1%)	68(85.0%)	205(91.9%)		
≥ 100 ml	30(9.9%)	12(15.0%)	18(8.1%)		
Blood transfusion	4(1.3%)	3(3.8%)	1(0.4%)	2.718	0.099
ICU observation	10(3.3%)	6(7.5%)	4(1.8%)	4.352	**0.037**
Total hospital stay (d)	16.85 ± 8.807	16.86 ± 8.045	16.85 ± 9.082	-0.013	0.990
Postoperative hospital stay	8.33 ± 5.006	8.43 ± 4.440	8.30 ± 5.203	-0.191	0.849
Remnant stone	13(4.3%)	4(5.0%)	9(4.0%)	0.002	0.965
Recurrent stone	4(1.3%)	2(2.5%)	2(0.9%)	0.257	0.612
Bile leakage	13(4.3%)	3(3.8%)	10(4.5%)	0.077	0.781
CBD stricture	6(2.0%)	1(1.3%)	5(2.2%)	0.006	0.937
Pancreatitis	0(0.0%)	0(0.0%)	0(0.0%)	0.000	1.000
Pneumonia	9(3.0%)	6(7.5%)	3(1.3%)	5.750	**0.016**
Acute cardiovascular event	4(1.3%)	4(5.0%)	0(0.0%)	7.787	**0.005**
Intra-abdominal hemorrhage	4(1.3%)	2(2.5%)	2(0.9%)	0.257	0.612
Fever	20(6.6%)	8(10.0%)	12(5.4%)	2.037	0.153
Urinary retention	1(0.3%)	1(1.3%)	0(0.0%)	2.797	0.094
30-day mortality	1(0.3%)	1(1.3%)	0(0.0%)	2.797	0.094

In both groups, no patients experienced pancreatitis. Three patients in Group A had bile leakage, while bile leakage occurred to 10 patients in Group B (3.8% vs. 4.5%, P = 0.781) (Table 3). At our center, most patients experiencing bile leakage were usually managed by drainage and lavage. However, for patients experiencing persistent bile leakage, a plastic stent would be inserted via endoscopic retrograde cholangiopancreatography (ERCP). All the patients experiencing bile leakage fully recovered after drainage and lavage. None of these 13 patients underwent reoperation to treat bile leakage. Regarding urinary retention, no significant differences between the two groups existed (1.3% vs. 0.0%, P = 0.094) (Table 3). Intra-abdominal hemorrhage occurred in 2 patients from Group A and 2 patients from Group B (2.5% vs. 0.9%, P = 0.612) (Table 3). Eight patients in Group A and 12 patients in Group B experienced postoperative fever (10.0% vs. 5.4%, P = 0.153) (Table 3). One patient in Group A experienced 30-day mortality, while no patients from Group B experienced 30-day mortality (1.3% vs. 0.0%, P = 0.094) (Table 3).

### Follow-up

During the subsequent follow-up, 2 patients from Group A experienced recurrence of calculus, while 2 patients from Group B experienced recurrence of calculus (2.5% vs. 0.9%, P = 0.612). One patient in Group A and 5 patients in Group B experienced stenosis of the CBD (1.3% vs. 2.2%, P = 0.937). For patients suffering from stenosis of the CBD, a 10 F biliary stent was implanted into the CBD immediately, and three months after implantation, the biliary stent was removed.

### Subgroup analysis excluding transcystic exploration

In order to better understand the safety of laparoscopic common bile duct exploration with primary closure among elderly patients (≥ 70 years), we then performed subgroup analysis where transcystic exploration had been excluded. Similarly, it was demonstrated that Group A was not significantly different from Group B except ICU observation (P = 0.039), pneumonia (P = 0.014) and acute cardiovascular event (P = 0.005). Comparisons between Group A and Group B had been presented in Table 4.

**Table 4:** Comparisons between Group A and Group B regarding postoperative results and follow-up outcomes after excluding transcystic exploration

Characteristics	No.	A	B	χ^2^/t	P
	(N = 278)	(N = 72)	(N = 206)	Value	Value
Operative time (min)	169.97 ± 69.26	173.90 ± 64.80	168.59 ± 70.85	-0.559	0.576
Estimated blood loss (mL)				2.907	0.088
<100 ml	250(89.9%)	61(84.7%)	189(91.7%)		
≥ 100 ml	28(10.1%)	11(15.3%)	17(8.3%)		
Blood transfusion	3(1.1%)	2(2.8%)	1(0.5%)	2.626	0.105
ICU observation	9(3.2%)	5(6.9%)	4(1.9%)	4.262	**0.039**
Total hospital stay (d)	16.96 ± 9.04	16.99 ± 8.29	16.95 ± 9.31	-0.032	0.975
Postoperative hospital stay	8.40 ± 5.18	8.44 ± 4.59	8.38 ± 5.38	-0.093	0.926
Remnant stone	13(4.7%)	4(5.6%)	9(4.4%)	0.007	0.931
Recurrent stone	4(1.4%)	2(2.8%)	2(1.0%)	0.285	0.594
Bile leakage	13(4.7%)	3(4.2%)	10(4.9%)	0.057	0.812
CBD stricture	6(2.2%)	1(1.4%)	5(2.4%)	0.003	0.959
Pancreatitis	0(0%)	0(0%)	0(0%)	0.000	1.000
Pneumonia	9(3.2%)	6(8.3%)	3(1.5%)	6.009	**0.014**
Acute cardiovascular event	4(1.4%)	4(5.6%)	0(0%)	8.024	**0.005**
Intra-abdominal hemorrhage	4(1.4%)	2(2.8%)	2(1.0%)	0.285	0.594
Fever	17(6.1%)	6(8.3%)	11(5.3%)	0.393	0.531
Urinary retention	1(0.4%)	1(1.4%)	0(0%)	2.870	0.090
30-day mortality	1(0.4%)	1(1.4%)	0(0%)	2.870	0.090

## Discussion

For patients with choledocholithiasis, LCBDE is one of the minimal treatment choices [2, 3]. Laparoscopic cholecystectomy plus CBDE enables surgeons to simultaneously solve two problems and allows patients to undergo general anesthesia only once with favorable results and a low complication rate. Conventionally, a T drainage tube was placed into the CBD for at least 2 weeks after surgery with the aim of decreasing pressure within the CBD, thus reducing the possibility of postoperative bile leakage and offering a backup percutaneous approach for cholangiography and extracting residual stones. However, a few complications related to T drainage tubes have been proposed, such as biliary obstruction, fluid and electrolyte disturbances, bile leakage and chronic pain. Additionally, stenosis of the CBD is also a complication after removal of the T drainage tube [20]. Bile leakage was completely avoidable even in the presence of a T drainage tube. Additionally, living with a T drainage tube for several weeks might lead to nonnegligible discomfort, more significant abdominal scarring, and burdens on mind and finances. All these aforementioned troubles related to T drainage tubes are likely to severely affect patients’ quality of life. In this new age of laparoscopy, treatment methods are becoming increasingly minimally invasive to reduce surgery-related trauma, enhance recovery and shorten hospital stay. Thus, T drainage tube placement seems to compromise the advantages of laparoscopic surgery.

Over the past few years, a series of studies comparing LCBDE with T drainage tubes and LCBDE without T drainage tubes have been published [3, 10–14, 17]. According to these studies, for carefully selected patients, primary closure of the CBD was feasible, safe and effective in treating patients with choledocholithiasis [3, 10–14, 17]. Thus, we could say that the era of routine T drainage tube insertion has ended.

Choledocholithiasis is a common disease among elderly patients. However, senior age will bring some considerable challenges: a high prevalence of comorbidities, such as pulmonary diseases, cardiovascular diseases, and diabetes mellitus; relatively poorer tolerance to surgical trauma and general anesthesia due to the decreased functional reserve of multiple organs; and significantly slower recovery after surgery. Studies reporting the feasibility, safety and efficacy of LCBDE plus primary closure of the CBD among elderly patients are still not abundant.

Thus, the present study was performed to further assess the feasibility, safety and efficacy of LCBDE plus primary closure of the CBD among elderly patients. In this study, elderly patients had a significantly higher incidence of concurrent diseases, such as hypertension, diabetes, and cardiovascular diseases. Correspondingly, the ASA scores of elderly patients were significantly higher, implying that compared with younger patients, elderly patients faced remarkably higher risks for cardiopulmonary complications after general anesthesia. In fact, elderly patients in our study did have a significant incidence of pneumonia and acute cardiovascular events after laparoscopic surgery. Therefore, it was understandable that elderly patients were more likely to experience ICU stays. In terms of demographic and clinical variables, elderly patients were not significantly different from younger patients except age and history of cholecystectomy. Reassuringly, in this study, elderly patients were not significantly different from younger patients regarding operative time, estimated blood loss, blood transfusion, postoperative stay, 30-day mortality and most postoperative complications except for a higher incidence of pneumonia and acute cardiovascular events. Despite the fact that elderly patients were not significantly different from younger patients in terms of postoperative hospital stay, we tend to be more prudent when discharged elderly patients since they recovered more slowly than younger patients. Due to the progress made in anesthesiology and organ support technology, supervision and management of underlying diseases are becoming increasingly meticulous and efficient. For all the patients in this study, a comprehensive evaluation algorithm was adopted to fully assess physical status, especially for elderly patients. For elderly patients, electrocardiogram, echocardiography, pulmonary function test, chest radiograph or CT, liver function test and kidney function test were routinely performed to fully evaluate tolerance of elderly patients to laparoscopic surgery and general anesthesia. Additionally, examinations assessing severity of comorbidities would be performed to patients with corresponding comorbidities. Laparoscopic surgery was performed only if all the examinations mentioned above were at good levels. Therefore, both risks of organ dysfunction and incidences of postoperative complications were controllable and comparable to those of younger patients.

No significant differences regarding surgical technique-related results existed. These results included remnant stones, bile leakage, stenosis of the CBD and stone recurrence and they could directly reflect quality of laparoscopic common bile duct exploration with primary closure. Remnant stones were detected among 4 elderly patients, while 9 younger patients experienced remnant stones. All patients experiencing remnant stones suffered from multiple choledocholithiasis prior to surgery, which might explain their remnant stones. We speculated that small stones or smaller fragments of larger stones were flushed into upstream intrahepatic bile ducts and located in the blind spot of the choledochoscope. These stones migrate to the distal part of the CBD with bile excretion and eventually cause abdominal pain. Difficulties during LCBDE with primary closure were not significantly affected by senior age. With the continuously increasing number of laparoscopic surgeries, surgeons at our center have gained much more experience in hepatobiliary surgeries, including a more in-depth understanding of the anatomy of intrahepatic bile ducts, more reasonably selecting surgical approaches, controlling bleeding and managing unexpected intraoperative situations, all of which enable surgeons to perform more standardized and proficient surgeries. Therefore, it was no surprise for us that senior age did not significantly affect the results of patients after LCBDE with primary closure.

Despite the encouraging results of this study, studies opposing primary closure of CBD after LCBDE have been published. In a study by Cai et al., it was reported that primary closure of the CBD after LCBDE should not be performed among patients suffering from acute obstructive suppurative cholangitis (AOSC) or those with outlet stenosis of the CBD since these patients need continuous and long-term decompression and drainage [21]. Xie W et al. reported that laparoscopic common bile duct exploration with primary closure was safe and effective for choledocholithiasis [22]. However, T tube drainage was a safe alternative method for bile duct closure in certain special cases, such as acute cholangitis, large stones, impacted stones, and laser lithotripsy [22]. Therefore, for patients under these circumstances, T tube drainage should be considered as an alternative to primary closure. Similarly, Lu J et al. reported the superiority of primary closure over T tube drainage but also argued that T tube drainage should not be absolutely abandoned [23]. They thought that for patients suffering from acute obstructive suppurative cholangitis or stenosis of common bile duct, T tube drainage should be the preferred choice [23].

In our study, 3 elderly patients and 10 younger patients experienced bile leakage after primary closure of the CBD after LCBDE, but the difference was not significant. The thin wall of the CBD was likely to be one of the contributing factors to bile leakage. Apart from the thickness of the CBD, operation techniques and suturing are also factors affecting the incidence of bile fistula. It has also been suggested that an incision should be made at the point where the CBD, cystic duct and common hepatic duct converge since there were significantly less vascular distributions at this point, which could further reduce the incidence of injury to the bile duct caused by electrocoagulation for the hemorrhagic spot. Upon the completion of closure of the incision on the bile duct by interrupted or continuous suture, we routinely accomplished interrupted sutures on the surface. This technique would help decrease suture tension and could potentially reduce the risk of bile leakage. Additionally, patients suspected to have malignant biliary tumors or those who had undergone biliary surgeries were not recommended to undergo primary closure of the CBD.

This study was not prospectively randomized, suggesting that direct comparisons between elderly patients and younger patients were difficult to make. However, given that the elderly patients were not significantly different from younger patients in terms of most baseline characteristics except some common morbidities, comparisons between these two groups were fair. We should also state that surgeons must be quite prudent when making surgical plans, especially for surgeons who are not quite experienced in grasping indications and contraindications. Thus, surgeons should try their best to better grasp indications and contraindications and improve their surgical skills after undergoing intensive training in laparoscopic skills. Surgeons should attach more importance to these aspects since they will potentially improve the surgical outcomes of patients.

## Conclusions

Therefore, considering all the aforementioned results of this study, we may conclude that for selected elderly patients, laparoscopic common bile duct exploration plus primary closure of the CBD is feasible and could be safely performed. Senior age should not be considered an absolute contraindication preventing laparoscopic common bile duct exploration plus primary closure.

## Data Availability

The datasets used and/or analyzed during the current study are available from the corresponding author on reasonable request.

## List of Abbreviations

LCBDE: Laparoscopic common bile duct exploration
CBD: Common bile duct (CBD)
ERCP: Endoscopic retrograde cholangiopancreatography
MRCP: Magnetic resonance cholangiopancreatography
CT: Computed tomography
LC: Laparoscopic cholecystectomy
ICU: Intensive care unit
SD: Standard deviation
SPSS: Statistical Package for the Social Sciences
BMI: Body mass index
ASA: American Society of Anesthesiologists
PACS: Picture archiving and communication system
